# Comparative Immunogenicity of Inactivated H7N9 Avian Influenza Vaccines with Different Internal Gene Backbones

**DOI:** 10.3390/microorganisms14081719

**Published:** 2026-08-05

**Authors:** Yi Liu, Mengyuan Bai, Tao Zhang, Yunqi Cui, Xiaowen Du, Lihong Huang, Jiahao Zhang, Ming Liao, Wenbao Qi

**Affiliations:** 1 Key Laboratory of Zoonoses of Ministry of Agriculture and Rural Affairs, South China Agricultural University, Guangzhou 510642, China; liuyi19981022@163.com (Y.L.); sini@eastpoint.com.cn (M.B.); a1085264668@163.com (T.Z.); cuiyq118@163.com (Y.C.); lihohuang2@scau.edu.cn (L.H.); jiahaozhang@scau.edu.cn (J.Z.); 2 National Avian Influenza Para-Reference Laboratory, South China Agricultural University, Guangzhou 510642, China; 3 National and Regional Joint Engineering Laboratory of Medicament of Zoonoses Prevention and Control, South China Agricultural University, Guangzhou 510642, China; mliao@scau.edu.cn; 4 State Key Laboratory of Animal Disease Control and Prevention, South China Agricultural University, Guangzhou 510642, China; 5 Key Laboratory of Zoonoses Prevention and Control of Guangdong Province, South China Agricultural University, Guangzhou 510642, China; 6 College of Animal Science & Technology, Zhongkai University of Agriculture and Engineering, Guangzhou 510225, China; 15651895178@163.com

**Keywords:** avian influenza virus, H7N9, vaccine, reverse genetics

## Abstract

H7N9 avian influenza virus (AIV) poses a persistent threat to poultry and public health. Despite widespread vaccination in China, rapid antigenic drift and reassortment necessitate frequent updates of vaccine strains. Phylogenetic analysis of isolates from Chinese provinces (from 2019 to 2023) showed that while surface genes diversified, the internal gene cassette remained conserved yet actively reassorted with other subtypes, suggesting internal gene compatibility may influence vaccine performance. We selected the H7N9 strain A/chicken/Northeast China/19854-6/2019 (E2), which harbors a polybasic Hemagglutinin (HA) cleavage site and predicted dual receptor-binding affinity. To enable safe vaccine development, we modified the HA cleavage site to generate a low-pathogenicity strain (E2-Δ). Using reverse genetics, we constructed three recombinant viruses: E2-Δ (retaining contemporary internal genes), CVI-E2, and CVII-E2 (containing internal genes from commercially used donor strains CVI and CVII, respectively). Inactivated vaccines were evaluated in specific-pathogen-free (SPF) chickens. E2-Δ induced HI antibody titers comparable to those of CVI-E2 and significantly higher than those of CVII-E2, and showed modestly higher cross-reactive HI titers against some recent H7N9 variants in exploratory analyses. All vaccines provided complete homologous protection with reduced viral shedding and no clinical signs in challenge trials. Our findings suggest that internal gene backbone compatibility may influence vaccine immunogenicity. While E2-Δ outperformed CVII-E2, it was comparable to CVI-E2, indicating that certain traditional backbones may still be suitable for H7N9 vaccine development. This approach warrants further validation to support a refined vaccine design strategy for H7N9 and potentially other avian influenza subtypes.

## 1. Introduction

Since its first emergence in 2013, the H7N9 subtype AIV has not only caused significant economic losses to China’s poultry industry but also posed a continuous threat to global public health security due to its cross-species transmission potential and the high mortality rate associated with human cases [1]. HA proteins of the H5 and H7 subtypes can convert from low pathogenicity to high pathogenicity by acquiring basic amino acids at the cleavage site. In 2016, emerging H7N9 subtype AIVs exhibited multiple motifs at the HA cleavage site, such as PKRKRTA(R/G), PKGKRTA(R/G), and PKGKRIA(R/G) [2]. Subsequently, H7N9 low pathogenic AIVs (LPAIVs) and highly pathogenic AIVs (HPAIVs) cocirculated in the poultry of affected area [3]. The H7N9 subtype AIV exhibits an evolutionary pattern characterized by “regional persistence coupled with phased virulence escalation.” The virus has circulated continuously within the poultry–live bird market ecosystem and has undergone multiple rounds of phylogenetic clade replacement and antigenic renewal [4]. During 2013–2017, H7N9 experienced five epidemic waves, during which LP-H7N9 mutated into HP-H7N9 in poultry, causing poultry mortality to surge to 70–100%, resulting in massive poultry deaths and human infections. A cumulative total of 1568 human cases and 616 deaths were reported [5]. Although human cases have not been reported since 2017, surveillance data indicate that the virus has not been eradicated from poultry populations and continues to evolve [6]. Vaccination remains one of the most effective measures for preventing and controlling avian influenza. Early epidemic strains were primarily divided into two major lineages: the Yangtze River Delta lineage and the Pearl River Delta lineage. Throughout subsequent waves of outbreaks, these strains continuously underwent genetic reassortment and shifts in dominant populations [7]. Consequently, a persistent challenge in controlling H7N9 is its rapid antigenic drift and active genetic reassortment, which lead to a swift decline in the match between vaccine strains and circulating field viruses [8,9]. In terms of genetic background, the internal genes of the H7N9 virus have long been derived from the H9N2 virus gene pool and underwent continuous genetic reassortment during circulation [10]. Studies have shown that its internal gene segments, including PB2, PB1, PA, NP, M, and NS, can all be traced back to H9N2 lineage origins. This reassortment pattern of “surface antigen renewal coupled with internal gene sharing” has enhanced the ecological adaptability of the virus [11,12].

Inactivated avian influenza vaccines are currently the most widely used and technically mature vaccine type for poultry influenza prevention and control worldwide, and they play a particularly central role in China’s compulsory immunisation policy against H5 and H7 subtype HPAI [13]. In September 2017, China launched a large-scale vaccination programme in poultry using H5/H7 bivalent inactivated vaccines [14]. This intervention effectively eliminated human H7N9 infections, no human cases have been reported since February 2019, and significantly reduced viral loads in poultry [15]. However, the virus has not been eradicated from poultry populations and continues to circulate, particularly in laying hens [16]. Between 2017 and 2019, under vaccination pressure, H7N9 underwent accelerated evolution: highly pathogenic strains gradually became dominant, while the prevalence of low-pathogenic strains declined. In early 2019, novel H7N9 viruses re-emerged in northeastern China and continued to circulate, forming an independent phylogenetic clade with substantial genetic distance from the 2013–2018 viruses [17]. Surveillance from 2020 to 2023 reported that 16 H7N9 viruses were isolated from samples collected across multiple Chinese provinces. Genetic analysis indicated that all these viruses belonged to a single genotype previously detected in poultry, with no evidence of reassortment with other subtypes. Notably, antigenic analysis revealed that while 12 of the 16 viruses remained antigenically close to the H7-Re4 (PR8 backbone) vaccine strain introduced in January 2022, four viruses showed reduced reactivity with the vaccine, indicating ongoing antigenic drift [6]. Consequently, the continuous evolution of H7N9 necessitates frequent updates of vaccine strains.

Current vaccine development relies primarily on reverse genetics, which generates candidate vaccine strains by reassorting the haemagglutinin (HA) and neuraminidase (NA) genes from circulating field strains with adapted, high-growth, and safe internal gene backbones (i.e., the six gene segments other than HA and NA)—most commonly A/Puerto Rico/8/1934 (PR8, designated CVII in this study) for both human and poultry vaccines [18]. For poultry vaccines, strain updates have followed the virus’s evolutionary trajectory: early vaccines using H7-Re1 (PR8 backbone) and H7-Re2 (PR8 backbone) strains exhibited low HI titres against 2019 isolates; subsequently updated H7-Re3 (PR8 backbone) and rLN79 (D7 backbone, designated CVI in this study) strains showed higher titres against 2019 viruses [17]. However, in early 2021, approximately six months after the introduction of H7-Re3 and rLN79 vaccine strains, novel H7N9 variants capable of escaping vaccine-induced immunity were detected in live poultry markets across Yunnan, Hebei, Shanxi, and Guangdong provinces. Studies have confirmed that these vaccines had insufficient protective capacity against the emerging variants under experimental conditions [19]. More recently (between 2021 and 2023), H7N9 viruses continued to evolve into new phylogenetic branches (including Group y.2.2, y.2.3, and y.2.4), with significant antigenic differences observed between branches [16]. Although reverse genetics has been successfully applied in developing various influenza vaccines, the backbone selection for commercial vaccine strains has remained relatively conservative. Accumulating evidence suggests that the compatibility between different internal gene backbones and surface glycoproteins may influence the biological characteristics and immunogenicity of the final vaccine strain [20,21]. However, existing vaccine development efforts have predominantly focused on surface antigen variation and updating, often overlooking the potential impact of the internal gene backbone on immunogenicity. Regarding the backbones mentioned above, the PR8 internal genes share low sequence homology with those of contemporary avian influenza viruses, potentially leading to suboptimal T-cell responses and impaired presentation of conformational HA epitopes [22,23,24,25]. In addition, vaccines based on the PR8 backbone present a narrower antigenic spectrum, as they lack internal proteins such as nucleoprotein (NP) and matrix protein 1 (M1) from circulating strains—proteins known to induce cross-reactive non-neutralising antibodies and T-cell responses, thereby contributing to broad protection [25,26,27]. Furthermore, it is noteworthy that since 2018, a dominant internal gene cassette has been established in highly pathogenic H7N9 viruses, meaning that the viruses no longer reassort with H9N2 but instead maintain a relatively stable set of internal genes. This phenomenon suggests that the internal gene constellation of H7N9 has undergone adaptive selection [28]. Therefore, screening for more suitable vaccine backbones is crucial for responding to the emergence of novel H7N9 epidemic strains and for future prevention and control efforts.

In this study, we selected the H7N9 strain A/chicken/Northeast China/19854-6/2019 (E2) as a model strain. This isolate possesses a polybasic HA cleavage site characteristic of highly pathogenic AIVs and exhibits molecular features associated with dual receptor-binding capacity, making it a relevant representative of recent field strains. To enable safe vaccine development, we modified the HA cleavage site to generate a low-pathogenicity backbone (E2-Δ). Using reverse genetics, we constructed three recombinant viruses: E2-Δ (retaining the contemporary internal genes of E2), CVI-E2, and CVII-E2 (containing the internal gene backbones of two commercial vaccine donor strains, CVI and CVII, respectively). We systematically evaluated the immunogenicity and protective efficacy of inactivated vaccines prepared from these recombinants in a SPF chicken model. Our objective was to determine whether the internal gene backbone compatibility of H7N9 AIV influences vaccine potency and to explore whether retaining a contemporary internal gene constellation could provide a novel strategy for optimizing H7N9 vaccine development.

## 2. Materials and Methods

### 2.1. Viruses and Cells

The viruses used in this study, including A/duck/Guangdong/D7/2007 (H5N2, CVI virus), A/Puerto Rico/8/1934 (H1N1, CVII virus), A/chicken/East China/H7SD12/2019 (SD12), A/chicken/East China/G285/2019 (G285), A/chicken/Northeast China/19376-E5/2019 (E5), A/chicken/Northeast China/19854-6/2019 (E2), A/chicken/Yunnan/21744/2021 (21744), A/chicken/Tianjin/22393/2022 (22393), and A/chicken/Hebei/22526/2022 (22526) were isolated from routine influenza surveillance. All viruses were propagated in 10-day-old specific pathogen-free (SPF) embryonated chicken eggs at 37 °C and frozen at −80 °C.

All cells used, including human embryonic kidney (HEK293T) cells and Madin-Darby canine kidney (MDCK) cells were provided by the National Avian Influenza Para-Reference Laboratory (Guangzhou, China) at South China Agricultural University. HEK293T and MDCK were grown in Dulbecco’s Modified Eagle’s Medium (DMEM) supplemented with 10% fetal bovine serum (FBS), 100 U/mL penicillin, and 0.1 mg/mL streptomycin and were incubated at 37 °C in a 5% CO_2_ incubator.

### 2.2. Plasmid Construction and Reverse Genetics

Viral RNA from the AIV isolates was extracted using the Viral RNA Extraction Kit (Fastagen, Shanghai, China, Catalog no. 220011). The RNA was reverse-transcribed into cDNA using the Reverse Transcriptase M-MLV (Takara, Kyoto, Japan, 2640A), according to the manufacturer’s guidelines. Using cDNA as a template, each target gene segment was amplified by PCR using the designed primers and Hoffmann’s avian influenza universal primers. The size of the PCR-amplified products was verified on a 1% agarose gel. After verification, the samples were subjected to sanger sequencing using an ABI 3730 sequencer (Sangon, Shanghai, China). The eight gene segments from the E2 strain were cloned into Hoffmann’s bidirectional transcription vector pHW2000 [18]. The primers used for PCR are listed in the Table S1. The entire genomes of all rescued viruses (E2-WT, E2-Δ, CVI-E2, CVII-E2) were sequenced using Sanger dideoxy sequencing. For each segment, RT-PCR products were sequenced in both directions. We compared the consensus sequences with the parental E2 strain and the donor backbone sequences. No unintended mutations were observed in any of the rescued viruses; the HA deletion was confirmed in E2-Δ, CVI-E2, and CVII-E2. During virus propagation in embryonated chicken eggs, E2-Δ, unlike E2-WT, did not cause embryo mortality.

HEK293T cell monolayers in 12-well plates were transfected at 80–90% confluency with 2.4 μg of the eight plasmids (300 ng of each plasmid) using Lipofectamine 2000 (Invitrogen, Carlsbad, California, USA), in line with the manufacturer’s instructions. DNA and transfection reagent were mixed and incubated at room temperature for 20 min and then added to the cells. Four hours later, the medium was replaced with Opti-MEM (GIBCO) containing 0.2% bovine serum albumin (BSA). After 48 h, the supernatant was harvested and inoculated into SPF embryonated chicken eggs for virus propagation. The rescued H7N9 viruses were confirmed using RT-PCR and sequencing.

### 2.3. Phylogenetic Analysis

The complete coding sequences for the influenza viruses genomes were obtained from the GISAID EpiFlu^TM^ database (accession IDs are listed in Supplementary Table S2). All data usage complies with the GISAID Terms of Use, including fair attribution to the submitting and originating laboratories. A dataset of viral gene sequences was compiled, which included publicly available sequences from GISAID and the H7N9 strains characterized in this study. Maximum Likelihood (ML) phylogenetic trees for the HA and NA gene alignments were inferred using IQ-TREE version 2.4.0 (http://www.iqtree.org/, accessed on 25 May 2026). The best-fit nucleotide substitution model was selected automatically by ModelFinder as implemented in IQ-TREE, which determined the GTR+F+G4 model to be optimal for both datasets based on the Bayesian Information Criterion (BIC). Node support was assessed using 1000 non-parametric bootstrap replicates. Phylogenetic trees with bootstrap values were visualized and annotated using FigTree (v1.4.4). The final trees were midpoint-rooted for illustrative purposes.

### 2.4. Hemagglutination (HA) Test

Add 25 µL of PBS buffer to all wells in columns A1–A12 of a 96-well V-bottom microtiter plate. Add 25 µL of chicken embryo allantoic fluid to column A1, mix by pipetting, then transfer 25 µL to column A2. Continue the two-fold serial dilution through to column A11, discarding 25 µL from the A11 well. Column A12 serves as the negative control. Add 25 µL of 1% fresh chicken erythrocytes (prepared in our laboratory) to each well, mix by gently tapping the plate, and incubate at room temperature for 30 min. Observe for hemagglutination.

### 2.5. Preparation of Inactivated H7N9 Immunogen

The four H7N9 subtype AIV strains were each serially diluted 10-fold in sterile PBS to a dilution of 10^−4^, and 0.2 mL of each dilution was inoculated into the allantoic cavity of 10-day-old embryonated chicken eggs. After incubation at 37 °C for 72 h, the allantoic fluid was harvested from each egg. An equal volume of the harvested allantoic fluid from each strain (before inactivation, allantoic fluids containing each virus strain were diluted to standardize to an equal HA titer of 7 log_2_ using sterile PBS as the diluent) was taken, and formaldehyde was added to a final concentration of 0.1% (*v*/*v*). After thorough mixing, the mixture was placed in a 37 °C incubator for inactivation for 24 h. After 24 h of inactivation, the antigen titer was tested and should remain unchanged (HA titer of 7 log_2_). Meanwhile, the inactivated allantoic fluid was inoculated into 10-day-old embryonated chicken eggs at 0.2 mL per egg. During incubation at 37 °C, embryo mortality was monitored periodically. After 72 h of culture, the allantoic fluid was tested by HA assay to determine its titer and verify the inactivation efficacy. If no hemagglutination titer was detected, complete inactivation was confirmed.

After passing the inactivation verification, vaccine preparation was carried out. Tween-80 (Solarbio, Beijing, China, 9005-65-6) was added to the inactivated antigen to a final concentration of 3.5% (prepared at a ratio of 700 μL Tween-80 per 5 mL antigen), vortexed to mix, and set aside. Meanwhile, the oil phase was prepared by thoroughly mixing 94 parts of Marcol-52 white mineral oil (PINRAN, Shanghai, China) with 6 parts of Span-80 (Macklin, Shanghai, China, 1338-43-8), which was then autoclaved and set aside for later use. A 10 mL aliquot of the oil phase was emulsified at 10,000 rpm for 5 min, after which the prepared antigen-Tween mixture was added to the oil phase. The speed was then increased to 17,000–20,000 rpm, and emulsification continued for 7 min until a homogeneous milky white emulsion was obtained [29]. After emulsification, the emulsion type was tested: a small amount of the vaccine was drawn with a clean Pasteur pipette and dropped into cold water to observe whether it dispersed. Sterility testing was also performed by inoculating the prepared vaccine into culture medium and observing any bacterial growth.

Inactivated vaccines were prepared from four rescued recombinant viruses: E2-WT (intact HPAIV, serving as a cleavage-site control), E2-Δ, CVI-E2, and CVII-E2. All procedures involving live E2-WT virus, including propagation and inactivation, were performed in the ABSL-3 facility. For the HA-deletion constructs (E2-Δ, CVI-E2, CVII-E2), propagation and inactivation were conducted under BSL-2 conditions after cleavage-site modification. Complete inactivation was confirmed by three blind passages in embryonated eggs with no detectable haemagglutination activity, after which all inactivated antigens were handled for emulsification. The E2-WT, E2-Δ and other HA-deletion constructs were used for vaccine preparation, and that all work with intact HPAIV was confined to ABSL-3.

### 2.6. Animal Immunization, Serum Collection and Hemagglutination Inhibition (HI) Assay

A total of 50 six-week-old SPF chickens were selected and divided into 5 groups (10 chickens per group), consisting of 4 experimental groups and 1 control group. The prepared inactivated vaccines were administered intramuscularly at a dose of 0.2 mL, while the control group received an injection of 0.2 mL sterile PBS. At weeks 2 and 3 post-immunization, blood was collected from the wing vein of chickens in both the vaccine groups and the PBS control group, serum was separated, and antibody levels were measured by HI test. The challenge experiment was conducted 21 days after vaccination of the SPF chickens. The E2-WT strain was used for challenge via the oculonasal route at a dose of 106 EID_50_ per bird in 200 μL (100 μL per eye and 100 μL intranasally). Oropharyngeal and cloacal swabs were collected from the SPF chickens in each group on days 3, 5, and 7 post-challenge. The collected swabs were stored in 1 mL of sterile PBS containing penicillin and streptomycin. Each swab sample was processed and inoculated into three 9- to 11-day-old embryonated chicken eggs, which were candled every 24 h, and after 60 h of observation, the HA titer of the allantoic fluid was determined for all eggs. The mental state, feeding, and activity of the SPF chickens were observed, and the survival rate of each group was monitored continuously for 10 days. Clinical monitoring: chickens were observed daily for depression, anorexia, edema, cyanosis, and mortality. The experimental groups were reared in separate isolators in an environmentally controlled facility, and food and water were provided ad libitum. The experiment was conducted under controlled conditions with a temperature of 25 ± 0.3 °C, a relative humidity of 60%, and a 12 h photoperiod. At 3 days post-challenge, three chickens from each vaccinated group were randomly selected and euthanised for necropsy and lung virus titration; the remaining seven birds per group continued to be monitored for clinical signs and swab collection through day 7 post-challenge. For the control group, swabs were collected from surviving birds at day 3; no survivors remained for later time points. Throat and cloaca swab specimens were collected from each experimental group. Each sample was placed in 1 mL of phosphate-buffered saline (PBS) containing penicillin (2000 U/mL) and streptomycin (2000 U/mL) before being centrifuged at 10,000× *g* for 5 min. The resulting supernatant was inoculated into the allantoic cavity of 10-day-old specific pathogen-free embryonated chicken eggs and incubated for 48 to 72 h at 37 °C. All the experiments were conducted in a biosafety level 3 laboratory. All animals in the experiment were handled in accordance with the principles of the Basel Declaration and recommendations of the approved guidelines of the Experimental Animal Administration and Ethics Committee of South China Agriculture University. The animal study protocol was approved by the Ethics Committee of Institutional Animal Care and Use Committee at SCAU (2024-14, 5 November 2024).

Hemagglutination inhibition (HI) assays were then performed against the H7N9 viruses using chicken erythrocytes according to standard methods. Briefly, 4 hemagglutinating units (HAU) of the viral antigen were dispensed into each well of a 96-well plate. Chicken antisera were heat-inactivated at 56 °C for 30 min prior to the assay. Twofold serial dilutions of the inactivated antisera were then prepared and mixed with the viral antigen. The HI titer was defined as the reciprocal of the highest serum dilution that completely inhibited hemagglutination. A threshold titer of ≥16 was established to define a seropositive response. According to the World Organisation for Animal Health (OIE, now WOAH), HI antibody titers are accepted as a primary correlate of protection for inactivated influenza vaccines in poultry.

### 2.7. Virus Titration

MDCK cells on 96-well plates were infected with 10-fold serial dilutions of the viral stock. Three days post-inoculation, the infection situation of the cells was examined by immunocytochemistry. MDCK cells on 96-well plates infected with AIV were washed three times with PBS and fixed with pre-cold methanol for 30 min at −20 °C. Then, the cells were washed three times with PBS and incubated with primary antibody NP (prepared by our laboratory) for 1.5 h at RT, followed by the appropriate HRP-conjugated goat anti-mouse IgG (Jackson ImmunoResearch, West Grove, Pennsylvania, USA, 115-035-003) for 1 h at RT. Finally, the cells were chemically stained with the preparation mixture (1 mL 3-Amino-9-ethylcarbazole solution, 0.5 mL NaAc, 13.5 mL PBS and 15 μL 30% H_2_O_2_) for 30 min at RT and the number of infected wells were counted using a microscope. The 50% tissue culture infection dose (TCID_50_) of each sample was determined using the Reed and Muench method. For TCID_50_ determination, infected MDCK cells were visualized under an inverted light microscope (Nikon Eclipse TS100, Tokyo, Japan) at 200× magnification, and infected wells were counted visually by two independent researchers.

Determination of the 50% egg infectious dose (EID_50_): The virus is serially diluted 10-fold from 10^−1^ to 10^−10^. Inoculate eight embryonated chicken eggs per dilution, 0.2 mL per egg. Discard embryos that die within 24 h. After incubation at 37 °C for 48–72 h, harvest the allantoic fluid and titrate for hemagglutination. Calculate the EID_50_ using the Reed-Muench method.

All work with live H7N9 HPAIV (including E2-WT, challenge virus, and recombinant viruses before inactivation) was performed in an ABSL-3 facility.

### 2.8. Ethics Statement

All experiments involving H7 HPAIV were conducted in the animal biosafety level 3 (ABSL-3) laboratory in accordance with South China Agricultural University (SCAU) (CNAS BL0011) protocols. SPF chickens involved in experiments were reviewed and approved by the Institution Animal Care and Use Committee (IACUC) at SCAU and treated according to the guidelines (2017A002).

Humane endpoints definition (euthanasia when birds exhibited severe clinical signs including depression, anorexia, and neurological symptoms); monitoring frequency (twice daily); clinical scoring criteria; euthanasia method (cervical dislocation); and inter-observer reliability (two independent observers).

### 2.9. Statistical Analysis

All data are presented as the Mean ± 95% confidence interval (CI). One-way ANOVA was utilized to compare the difference between different groups. Statistical significance was indicated as * (*p* < 0.05), ** (*p* < 0.01), *** (*p* < 0.001), **** (*p* < 0.0001). Statistical significance was determined using GraphPad Prism 8.

## 3. Results

### 3.1. Whole-Genome Phylogenetic Analysis and Sequence Characterization of H7N9 Subtype Avian Influenza Viruses

**Figure 1:**
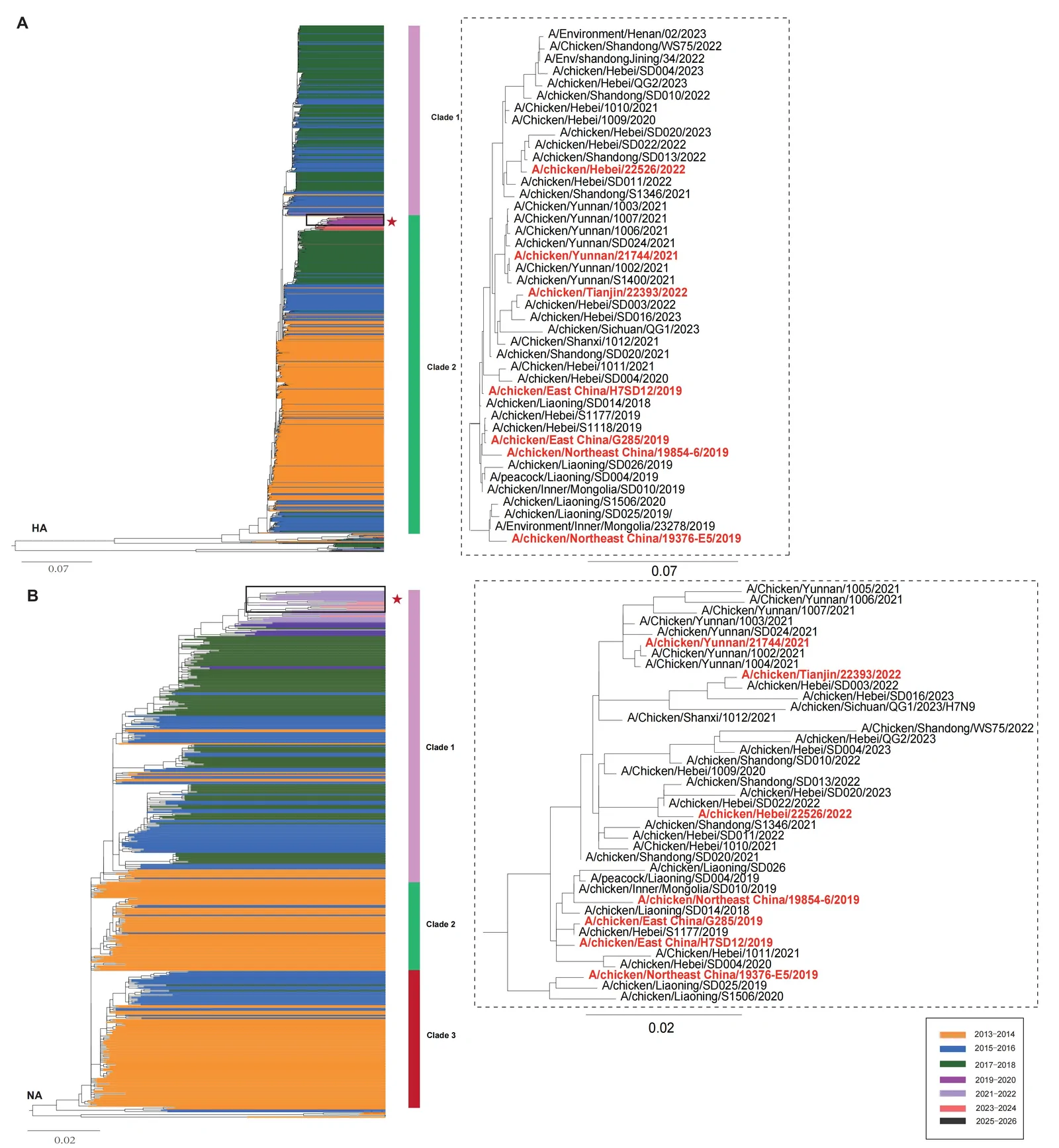
Phylogenetic analysis of HA and NA. Maximum likelihood (ML) phylogenetic trees of the HA (**A**) and NA (**B**) genes of H7N9 viruses. Except for the E2, SD12, G285, E5, 21744, 22393, and 22526 strains, all other virus sequences were downloaded from GISAID. Asterisks indicate the viruses characterized in this study. The full gene segments were used to construct an ML phylogenetic tree with IQ-TREE, using the GTR+F+G4 model, and the analysis was conducted with 1000 bootstrap replicates. The scale bar indicates branch length and corresponds to 0.07 (HA)/0.02 (NA) estimated amino acid substitutions per site.

**Figure 2:**
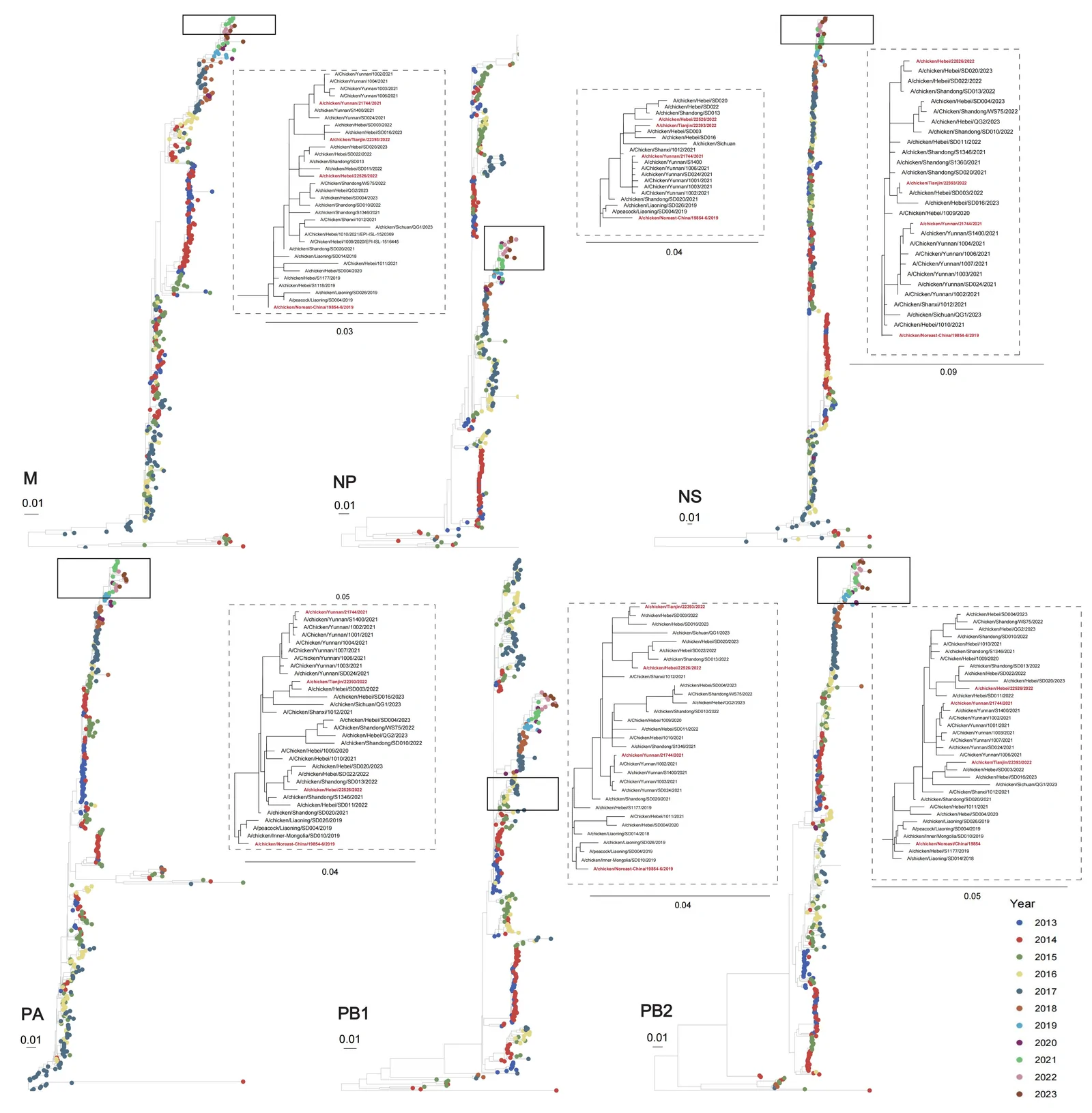
Phylogenetic analysis of other genes (PB1, PB2, PA, NP, M, and NS) of H7N9 AIV. Maximum likelihood (ML) phylogenetic trees of other genes (PB1, PB2, PA, NP, M, and NS) of H7N9 viruses. Except for the E2, 21744, 22393, and 22526 strains, all other virus sequences were downloaded from GISAID. The virus strains identified in this study are highlighted in red. The full gene segments were used to construct an ML phylogenetic tree with IQ-TREE, using the GTR+F+G4 model, and the analysis was conducted with 1000 bootstrap replicates.

To characterize the evolutionary history of H7N9 strains, we downloaded all whole-genome sequences of H7N9 subtype AIVs from GISAID, including A/chicken/Northeast China/19854-6/2019 (H7N9) (hereafter E2/H7N9) isolated in our laboratory. The genomic sequence of this strain have been submitted to the GISAID database (https://www.gisaid.org, accessed on 25 May 2026) (Table S2). Our findings reveal that H7N9 subtype AIVs were clustered together between 2013 and 2017. Following the implementation of the H7 vaccination strategy in China in September 2017, the isolation rate of H7N9 strains decreased significantly after 2018, although sporadic cases continued to occur. Phylogenetic analysis shows that the HA and NA gene of H7N9 viruses formed two and three clades, respectively. The clade 2 of HA gene and clade 1 of NA gene were widespread in poultry since 2019. In this study, we found that the HA and NA genes of the E2 strain were originated from the prevailing clade 2 and clade 1, respectively, indicating that E2 strain is predominant H7N9 strain in China. Specifically, we found that the HA and NA genes of E2 strain were genetically closely related to the H7N9 HPAIVs that persisted in northern China (i.e., Hebei, Inner Mongolia, and Liaoning provinces) during 2019 (Figure 1). Except for the surface genes, the internal genes of E2/H7N9 strain were also genetically closely related to the H7N9 strains that persist in Inner Mongolia, Liaoning, and Hebei provinces of China during 2019–2020 (Figure 2). In comparison with the genotypes (G1–G10) of H7N9 strains defined in previous studies [6], we found that E2/H7N9 strain is closely related to the G2 genotype. The G2 genotype belongs to the genotype of currently circulating strains. Geographically, this genotype has recently spread across both southern and northern China (i.e., Shandong, Yunnan, and Hebei provinces), exhibiting significant spatiotemporal representativeness. Together, these results indicate that the E2/H7N9 strain were widespread throughout China in recent years.

Molecular characterization of the E2/H7N9 strain revealed that this H7N9 strain was highly pathogenic avian influenza virus, which possessing the basic cleavage site motif PKRKRTTR↓GLF. Additionally, amino acid residues (H3 numbering [30,31]) of HA protein were also identified, including 160T, 186V, 226Q, and 228G substitutions. Previous studies have shown that the L226Q mutation of HA protein enhances the virus’s binding affinity for avian-type receptors [32]. The presence of 138A and 186V residues of HA protein were associated with human receptor binding capability, suggests that the strain possesses dual receptor-binding properties [33,34]. Analysis of potential N-linked glycosylation sites identified seven sites: ^30^NGT^32^, ^46^NAT^48^, ^141^NGT^143^, ^167^NAT^169^, ^249^NDT^251^, ^425^NWT^427^, and ^497^NNT^499^, which could be predominantly located in different domains of HA, including the head and stem regions, and their glycosylation is generally highly conserved. This conservation suggests that they play an indispensable role in maintaining the overall conformation of the HA protein and stabilizing its trimeric structure, potentially affecting the receptor-binding affinity of the virus [35,36]. Similarly, genetic analysis of the NA gene of this strain showed an open reading frame of 1398 nucleotides, encoding 465 amino acids. Analysis identified six potential N-linked glycosylation sites: ^42^NCS^44^, ^52^NTS^54^, ^63^NET^65^, ^82^NLT^84^, ^142^NGT^144^, and ^197^NAS^199^. These sites play critical roles in viral structural stability, replication efficiency, and immune evasion [35,37].

### 3.2. Reverse Genetics Rescue of Three H7N9 Recombinant Viruses with Distinct Backbones

**Figure 3:**
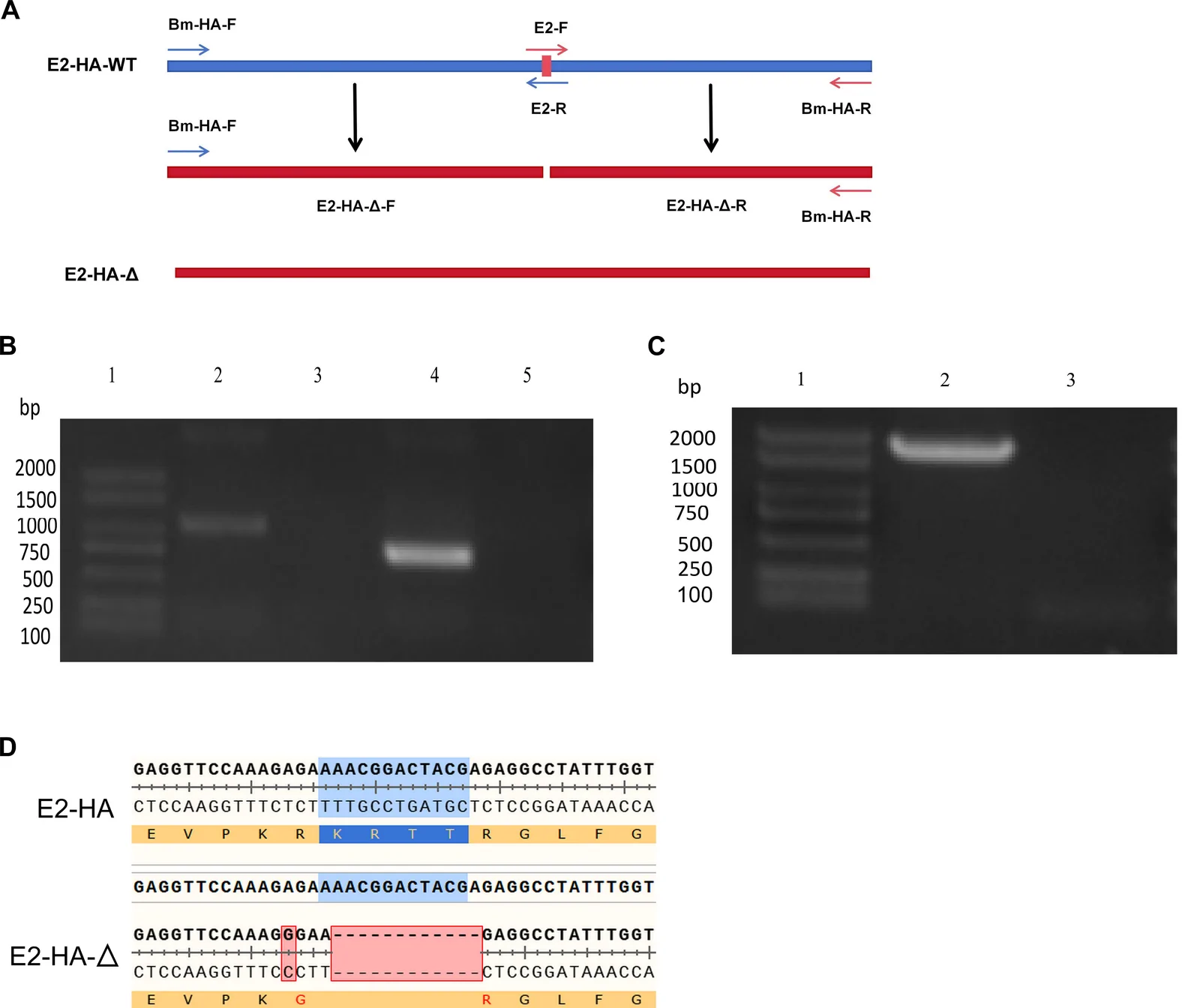
Construction of the sequence with a 4-amino-acid deletion at the cleavage site of the E2-HA gene (E2-HA-Δ). (**A**) Schematic diagram of the construction process of E2-HA-Δ. (**B**) Overlap PCR was used to delete four consecutive amino acids at the cleavage site of the HA gene. First, the upstream and downstream fragments flanking the basic cleavage site were amplified using the primer pairs Bm-HA-F/E2-R and E2-F/Bm-HA-R, yielding fragments of 1077 bp and 740 bp, respectively. The PCR amplification products were visualized by electrophoresis and photographed, recovered, and the samples verified as correct by sequencing were designated E2-HA-Δ-F and E2-HA-Δ-R, respectively, 1: Marker: Ladder 2000 plus; 2: E2-HA-Δ-F; 3: E2-HA-Δ-F negative control; 4: E2-HA-Δ-R; 5: E2-HA-Δ-R negative control. (**C**) Using Bm-HA-F and Bm-HA-R as primers, and E2-HA-Δ-F plus E2-HA-Δ-R as the template, the full-length HA gene with a 4-amino-acid deletion was amplified by fusion PCR, yielding a product of 1774 bp. The PCR product was recovered after electrophoresis, and the sample confirmed to be correct by sequencing was designated E2-HA-Δ, as shown in the right panel, 1: Marker: Ladder 2000 plus; 2: E2-HA-Δ; 3: E2-HA-Δ negative control. (**D**) Sequence alignment of E2-HA-WT and E2-HA-Δ (with a 4-amino-acid deletion at the HA gene cleavage site).

An eight-plasmid system was constructed by individually cloning the eight gene segments of the E2 strain. To eliminate the highly pathogenic signature and adapt it for vaccine development, the multi-basic amino acid cleavage site in the E2 HA gene was deleted (Figure 3A), yielding the modified gene designated HA-Δ. The original HA cleavage site (PKRKRTTR↓GLF) was mutated to PKGR↓GLF by deleting the four amino acids (Figure 3D). This leaves a monobasic cleavage site (R) that is typical of low-pathogenic avian influenza viruses and requires exogenous trypsin for replication in cell culture. Using reverse genetics, the HA-Δ and NA genes were then co-transfected into 293T cells together with the six internal genes derived from either the parental E2 strain or two commercial vaccine backbones, CVI and CVII (Table 1). After three consecutive passages in SPF chicken embryos, all rescued viruses showed stable hemagglutination (HA) titers of ≥7 log_2_. The full HA and NA genes were sequenced and no mutations were found in the modified cleavage site or elsewhere. The EID_50_ and TCID_50_ were determined for the four strains carrying the E2 surface genes (E2-WT, E2-Δ, CVI-E2, CVII-E2) (Table 1). All strains were therefore used in subsequent experiments.

### 3.3. Comparison of Humoral Immune Responses Induced by Inactivated H7N9 Vaccines with Distinct Internal Gene Backbones

**Figure 4:**
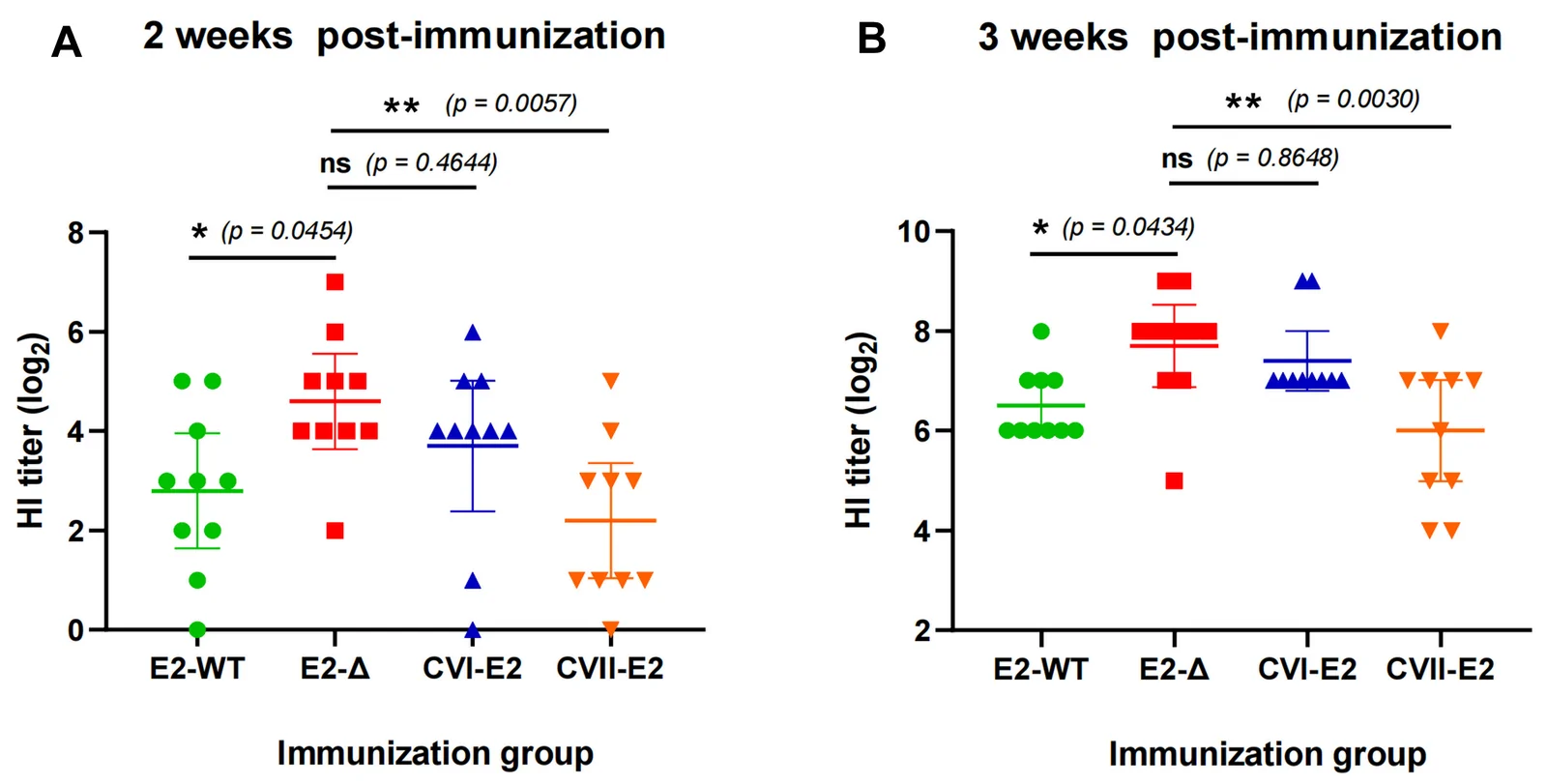
HI antibody titers of each vaccine group at weeks 2 (A) and 3 (B) post-immunization. At 2 (**A**) and 3 (**B**) weeks post-immunization, blood was collected from all SPF chickens (10 chickens per group) in the isolators, and sera were separated for HI assay using E2-WT as the antigen. All data are presented as the mean ± 95% confidence interval (CI). One-way ANOVA was utilized to compare differences between groups, followed by Dunnett’s post-hoc test for pairwise comparisons. Error bars indicate the 95% confidence interval (CI); ns (non-significant), * (*p* < 0.05), ** (*p* < 0.01).

The inactivated vaccines were prepared from the four rescued recombinant viruses (E2-WT, E2-Δ, CVI-E2, and CVII-E2). Serum samples were collected from immunized and negative-control SPF chickens via the wing vein at weeks 2 and 3 post-immunization, and HI assays were conducted to evaluate humoral immune responses. As shown in Figure 4A,B, at week 2 post-immunization, the mean HI titers (log_2_) were 2.8 for the E2-WT group, 5.0 for E2-Δ, 3.7 for CVI-E2, and 2.2 for CVII-E2. By week 3, antibody levels increased substantially in all groups, reaching 6.5 (E2-WT), 7.7 (E2-Δ), 7.4 (CVI-E2), and 6.0 (CVII-E2), with each group exceeding the commonly accepted protective threshold (HI titer ≥ 4 log_2_). Notably, the E2-Δ vaccine consistently elicited significantly higher HI titers than the E2-WT and CVII-E2 vaccines at both time points (*p* < 0.05), while no significant difference was observed compared with the CVI-E2 group.

**Figure 5:**
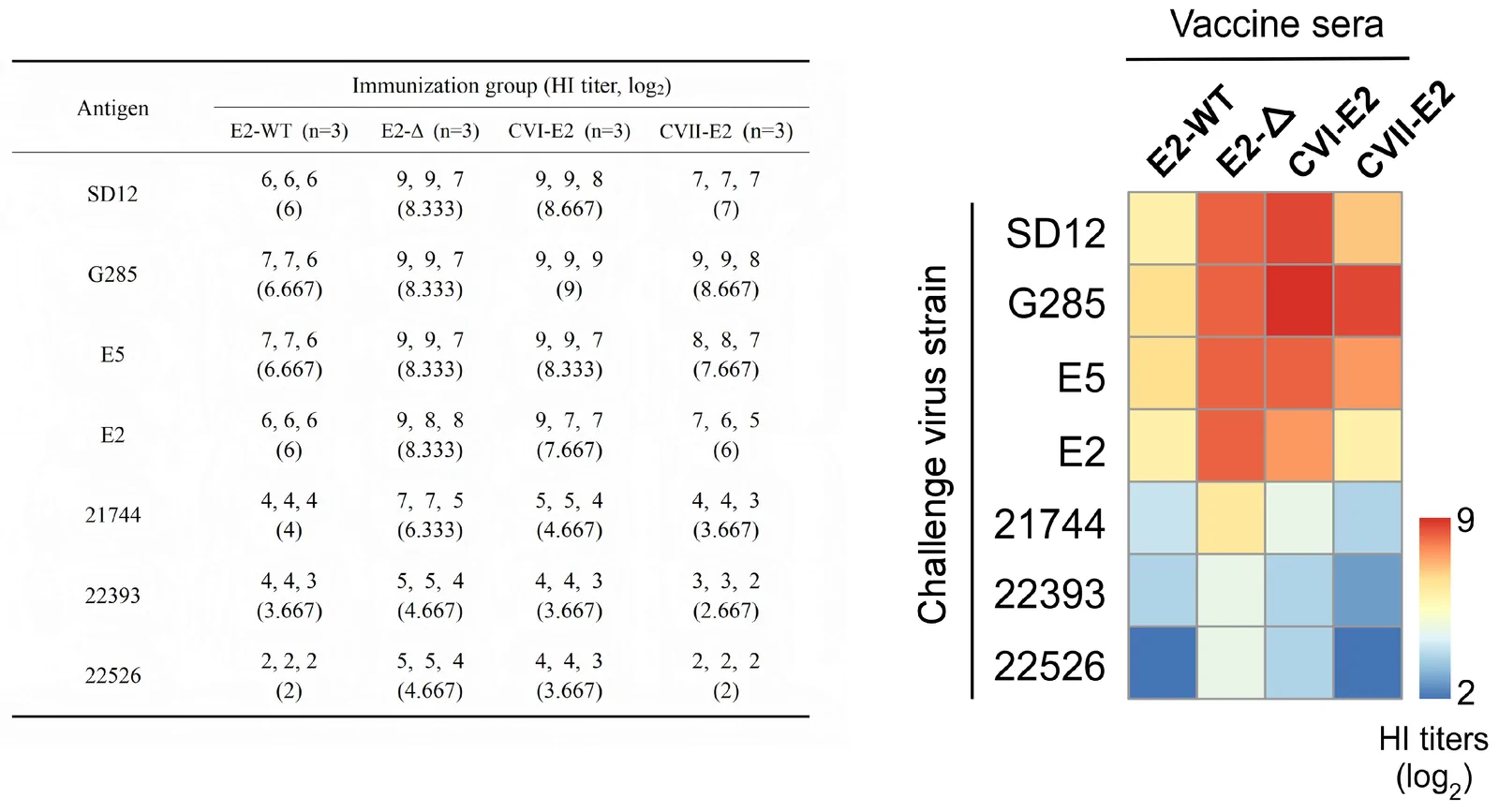
Results of HI cross-reactivity assay. Positive chicken sera collected at 3 weeks post-immunization from the groups immunized with inactivated vaccines E2-WT, E2-Δ, CVI-E2, and CVII-E2 were subjected to serological cross-reactivity assays against seven H7N9 subtype strains isolated from 2019 to 2022. The data are derived from the same serum samples as in Figure 4 but represent independent test results.

To assess the breadth of immune protection, cross-reactivity of sera collected at 21 days post-immunization was tested against seven H7N9 HPAIV strains isolated between 2019 and 2022 (Table 2). Sera from all vaccinated groups showed strong reactivity (HI titer ≥ 6 log_2_) against three 2019 isolates (strains SD12, G285, E5), confirming good antigenic conservation with earlier viruses. In contrast, reactivity against strains isolated in 2021 (strain 21744) and 2022 (strains 22393, 22526) was markedly reduced across all groups, with HI titers declining by 4- to 128-fold (Figure 5). This pattern suggests significant antigenic drift occurring in circulating H7N9 viruses after 2019.

Taken together, these results demonstrate that all candidate vaccines could induce protective antibody levels against the homologous strain. Among them, HI titers of the E2-Δ vaccine were comparable to those of CVI-E2 and significantly higher than those of CVII-E2. In addition, E2-Δ showed modestly improved cross-reactive antibody titers against some 2021–2022 variant strains compared with the other backbone constructs. However, as heterologous challenge studies were not performed, whether this serological advantage translates into enhanced cross-protection remains to be determined.

### 3.4. All Candidate H7N9 Inactivated Vaccines Confer Complete Clinical Protection Against Lethal Homologous Challenge in Spf Chickens

**Figure 6:**
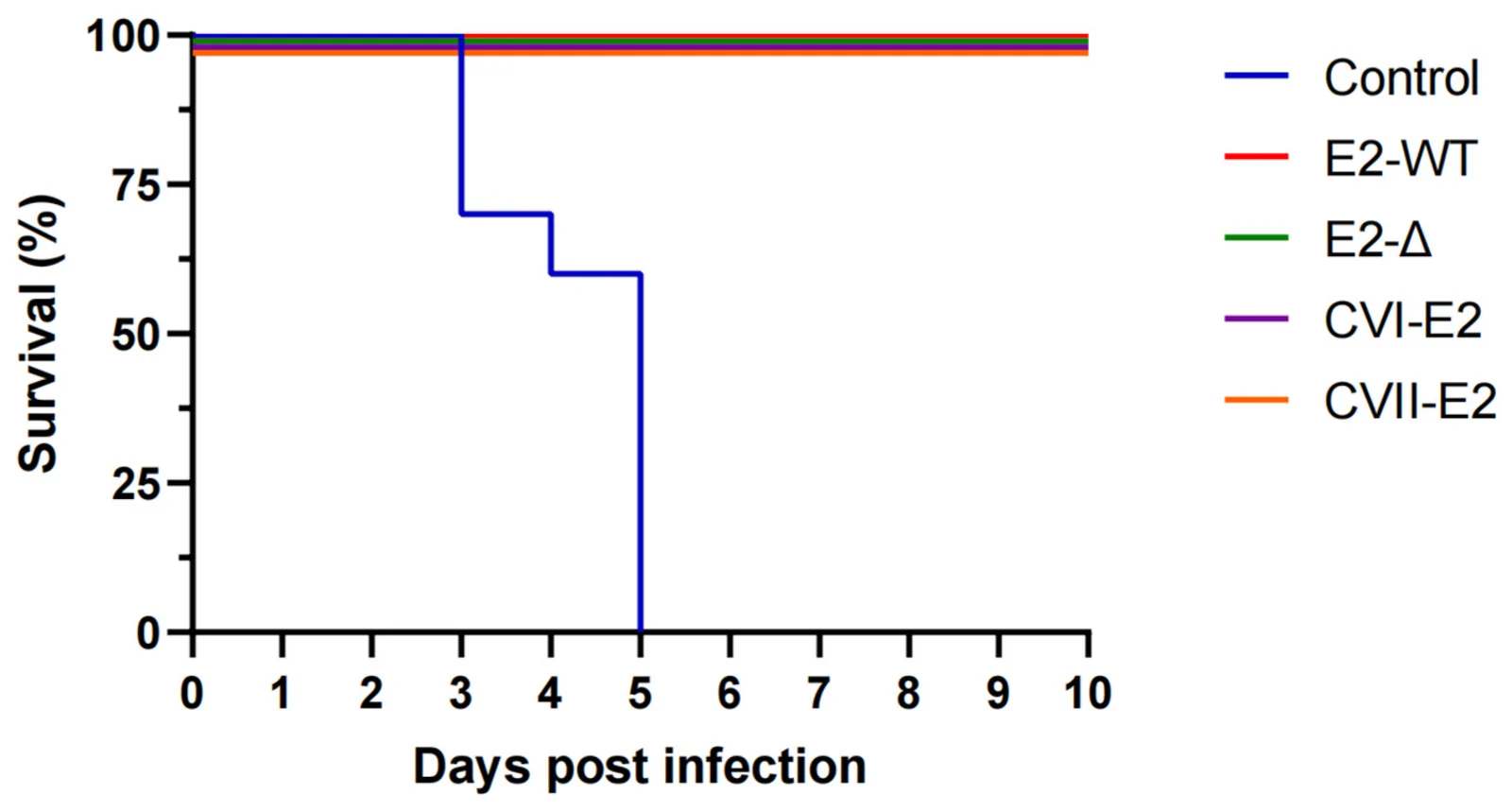
The SPF chickens were intranasally inoculated with E2 virus and monitored for 10 days for survival. At 3 weeks post-immunization, each SPF chicken in the immunized and control groups was challenged with the E2 strain (10^6^ EID_50_) and observed for 10 consecutive days to monitor survival rates. All vaccinated groups overlapped at 100% and the lines are superimposed.

At 21 days post-immunization, SPF chickens in all vaccinated groups (E2-WT, E2-Δ, CVI-E2, CVII-E2) and the negative control group were challenged intranasally with 10^6^ EID_50_ of the homologous A/chicken/Northeast China/19854-6/2019 (H7N9, E2) virus and monitored for 10 days. All vaccinated chickens survived until the end of the observation period without showing any clinical signs of disease. In contrast, control chickens developed severe clinical symptoms characteristic of highly pathogenic avian influenza, including depression, anorexia, hemorrhagic foot shanks, cyanotic and swollen combs, and facial edema. Mortality in the control group began on day 3 post-challenge and reached 100% by day 5 (Figure 6). Viral shedding was assessed via oropharyngeal and cloacal swabs collected on days 3, 5, and 7 post-challenge. While infectious virus was readily isolated from swabs collected from control group on day 3, no virus was detected in any swab samples from vaccinated chickens at all time points tested (Table 3). In addition, on day 3 post-challenge, the viral titers in the lungs of sampled chickens from each vaccinated group were zero, whereas viral replication was detectable in the lungs of the control group (Table S3).

Collectively, these results demonstrate that all vaccinated groups, including those receiving CVI-E2 and CVII-E2, conferred complete clinical protection against lethal homologous challenge, prevented detectable viral replication in respiratory tissues, and completely inhibited virus shedding from both respiratory and cloacal routes, with no clinical signs.

## 4. Discussion

Highly pathogenic H5Nx and H7N9 avian influenza viruses persistently threaten global poultry health and pose a significant zoonotic risk. Vaccination remains critical in China, but rapid antigenic evolution demands efficient and adaptable vaccine platforms. The continuous evolution of H7N9 AIV since its 2013 emergence in China has amplified its veterinary and public health significance. A pivotal shift occurred around 2016, when the insertion of a multi-basic amino acid motif at the HA cleavage site converted low-pathogenic to HPAI H7N9 viruses, drastically increasing their virulence in poultry [6,29,38,39,40]. Concurrently, the receptor-binding properties of these viruses have evolved. The model strain in this study, A/chicken/Northeast China/19854-6/2019, exemplifies this dual threat. It possesses the L226Q substitution in HA, known to strengthen binding to avian-type (α2,3-linked sialic acid) receptors [32,41]. It also carries the S138A and G186V mutations, associated with enhanced binding to human-type (α2,6-linked sialic acid) receptors. This trait may facilitate initial avian-to-human transmission and represents a critical step in viral adaptation to mammalian hosts [33,34,42]. Therefore, for vaccines targeting specific and persistently circulating virus lineages such as H7N9, selecting a highly matched internal gene backbone, in combination with typical HA attenuation modifications (e.g., cleavage site deletion), may represent one of the strategies to improve immunogenicity; however, further confirmation is still required.

In this study, the mechanistic basis for the observed differences in immunogenicity remains unclear and may involve multiple factors, including HA antigen conformation [43], glycosylation [44,45], membrane incorporation efficiency [46], or simply the temporal advantage of a more recent genetic background [47]. However, given the use of inactivated vaccines, it is unlikely that internal gene products such as NS1 directly modulate innate immunity in a functional manner. Future studies employing structural and immunological approaches are needed to elucidate the underlying mechanisms. This study is only a preliminary exploration of the impact of internal gene compatibility on vaccine immunogenicity, and there are still many limitations. First, the immunological evaluation primarily relied on HI antibody titers and challenge protection experiments; the contribution of cellular immune responses was not systematically assessed; and the HA folding and glycosylation processes have not been experimentally verified, which also constitutes a limitation. A limitation of this study is the exclusive focus on humoral immunity. Internal viral proteins encoded by the backbone genes (e.g., NP, M1, PB1, PA) are known to elicit cross-reactive CD8^+^ and CD4^+^ T-cell responses that contribute to heterosubtypic protection [48,49,50]. Future studies should assess NP- and M1-specific T-cell responses using IFN-γ ELISpot, intracellular cytokine staining, or lymphocyte proliferation assays to determine whether backbone compatibility influences cellular immunity. Cross-reactivity was assessed using a limited number of serum samples (*n* = 3 per group) as an exploratory analysis; larger sample sizes would be required to confirm these trends, and heterologous challenge protection experiments should also be performed. Therefore, any claim of cross-protection remains a hypothesis that requires further experimental validation. Second, vaccines were standardised by HA titre, which is the accepted method for antigen adjustment in poultry vaccine development and is recognised by WOAH and Chinese regulatory authorities for avian influenza vaccine quality control. While we acknowledge that HA titre measures functional haemagglutination activity rather than absolute HA protein mass (for which SRID is the gold standard), the absence of SRID reference reagents for the specific H7N9 strain used in this study precluded protein-based quantification. Nevertheless, standardising all vaccine groups to an equal HA titre ensured comparability of functional antigen content across backbone constructs. Future studies employing quantitative methods such as SRID or HA-specific ELISA would be valuable to confirm that observed immunological differences are not influenced by unequal HA protein dosing. Third, the yield of the vaccine strain in embryonated chicken eggs, its genetic stability, and compatibility with large-scale production processes were not explored in depth. Long-term stability over 10+ passages should be assessed before industrial use. Furthermore, the potential impact of the internal gene backbone on vaccine safety, such as the possibility of reversion to virulence through reassortment or mutation, requires long-term monitoring. Finally, The E2 strain served both as the source of contemporary internal genes and as the backbone for the E2-Δ vaccine construct. Consequently, any observed immunological advantage could arise from either the co-evolutionary compatibility of its own gene segments or simply from the temporal advantage of a more recent genetic background (2019) compared to the older CVI (2007) and CVII (1934) backbones. Our study was not designed to disentangle these two factors, and therefore our conclusions are limited to a strain-specific comparison rather than a generalizable principle of backbone superiority. Future studies should use reciprocal reassortants or time-calibrated backbones to address this confound.

## 5. Conclusions

In conclusion, this study demonstrated that using the H7N9 strain’s own internal gene backbone, instead of the CVII backbone (PR8), moderately improved the HI titers elicited by the inactivated vaccine. However, the advantage over the CVI backbone was not statistically significant, indicating that backbone selection should be carefully evaluated on a strain-by-strain basis. Whether this improved immunogenicity translates into broader cross-protection against antigenically drifted H7N9 strains remains to be determined in future heterologous challenge studies. Preserving the native internal gene constellation represents a potential alternative strategy, but not necessarily an optimized one, for H7N9 vaccine design. Ultimately, controlling H7N9 influenza requires an integrated strategy: leveraging insights into gene constellation compatibility for better vaccine design, while maintaining a dynamic surveillance system to guide the frequent updating of vaccine antigens in response to ongoing viral evolution.

## Figures and Tables

**Table 1:** Information concerning rescued virus strains.

Strain	HA	NA	Backbone Genes	HA Titer (log_2_)	EID_50_/mL	TCID_50_/mL
E2-WT	E2-HA	E2-NA	E2	7	1.78 × 10^9^	2.1 × 10^7^
E2-Δ	E2-HA (lacking KRTT)	E2-NA	E2	8	5.62 × 10^8^	1.6 × 10^7^
CVI-E2	E2-HA (lacking KRTT)	E2-NA	CVI	8	3.16 × 10^9^	3.2 × 10^7^
CVII-E2	E2-HA (lacking KRTT)	E2-NA	CVII	7	1.78 × 10^8^	2.1 × 10^7^

Note: All viruses were sequenced at passage 3 in embryonated eggs. No unintended mutations were introduced during the reverse-genetics rescue or subsequent egg propagation. The HA cleavage-site deletion was confirmed in all HA-deletion constructs, and no mutations were found in any other gene segments.

**Table 2:** Names and sources of virus strains used in the experiment.

Virus Strain	Abbreviation	Subtype	Year
A/chicken/East China/H7SD12/2019	SD12	H7N9	2019
A/chicken/East China/G285/2019	G285	H7N9	2019
A/chicken/Northeast China/19376-E5/2019	E5	H7N9	2019
A/chicken/Northeast China/19854-6/2019	E2	H7N9	2019
A/chicken/Yunnan/21744/2021	21744	H7N9	2021
A/chicken/Tianjin/22393/2022	22393	H7N9	2022
A/chicken/Hebei/22526/2022	22526	H7N9	2022

**Table 3:** Virus shedding in SPF chickens.

Group	Throat Swab			Cloacal Swab		
	3 d	5 d	7 d	3 d	5 d	7 d
E2-WT	0/7	0/7	0/7	0/7	0/7	0/7
E2-Δ	0/7	0/7	0/7	0/7	0/7	0/7
CVI-E2	0/7	0/7	0/7	0/7	0/7	0/7
CVII-E2	0/7	0/7	0/7	0/7	0/7	0/7
Control	7/7	-	-	7/7	-	-

Note: “-“ indicates that all chickens in the group had died by that time point. At 3 days post-challenge, 3 chickens from each vaccinated group were randomly selected and euthanised for lung virus titration (Table S3); the remaining 7 chickens were sampled for swabs at 5 and 7 dpc. For the control group, only 7 birds survived to day 3 (3 had died by then), and all were virus-positive; no chickens survived to day 5 or 7. Throat and cloacal swab samples were identified by virus isolation using SPF embryonated eggs.

## Data Availability

This paper does not report original code. Any additional information required to reanalyze the data reported in this paper is available from the lead contact upon request. All data generated and/or analysed during this study are included in this article and its supplementary information files. Raw HI titres, phylogenetic alignments (http://purl.org/phylo/treebase/phylows/study/TB2:S32720?x-access-code=68acbe49cd81a87f093bb2f4cfd56067&format=html, accessed on 25 July 2026), and additional virological data are available in the Supplementary Materials. The E2 strain genome sequences have been deposited in the GISAID database under accession numbers listed in Table S2.

## Supplementary Materials

The following supporting information can be downloaded at https://www.mdpi.com/article/10.3390/microorganisms14081719/s1: Figure S1: Comparative Immunogenicity of Inactivated H7N9 Avian Influenza Vaccines with Different Internal Gene Backbones; Figure S2. Phylogenetic analysis of other genes (PB1, PB2, PA, NP, M, and NS) of H7N9 AIV (from Figure 2); Table S1: Primers used for the rescue of avian influenza virus by reverse genetics; Table S2: The accession numbers in GISAID of E2 (H7N9) strains in this study; Table S3: Viral titers in the lung tissues of SPF chickens on day 3 post-challenge in each group; Table S4. HI antibody titers of each vaccine group at weeks 2 and 3 post-immunization.
