# Supplementary material for: Comparative Immunogenicity of Inactivated H7N9 Avian Influenza Vaccines with Different Internal Gene Backbones
Source: Microorganisms. 2026 Aug 5;14(8):1719. doi: 10.3390/microorganisms14081719 (PMC13515558; doi:10.3390/microorganisms14081719)
Supplement: Supplementary file 1 [file microorganisms-14-01719-s001.zip › microorganisms-4413796-supplementary.pdf]

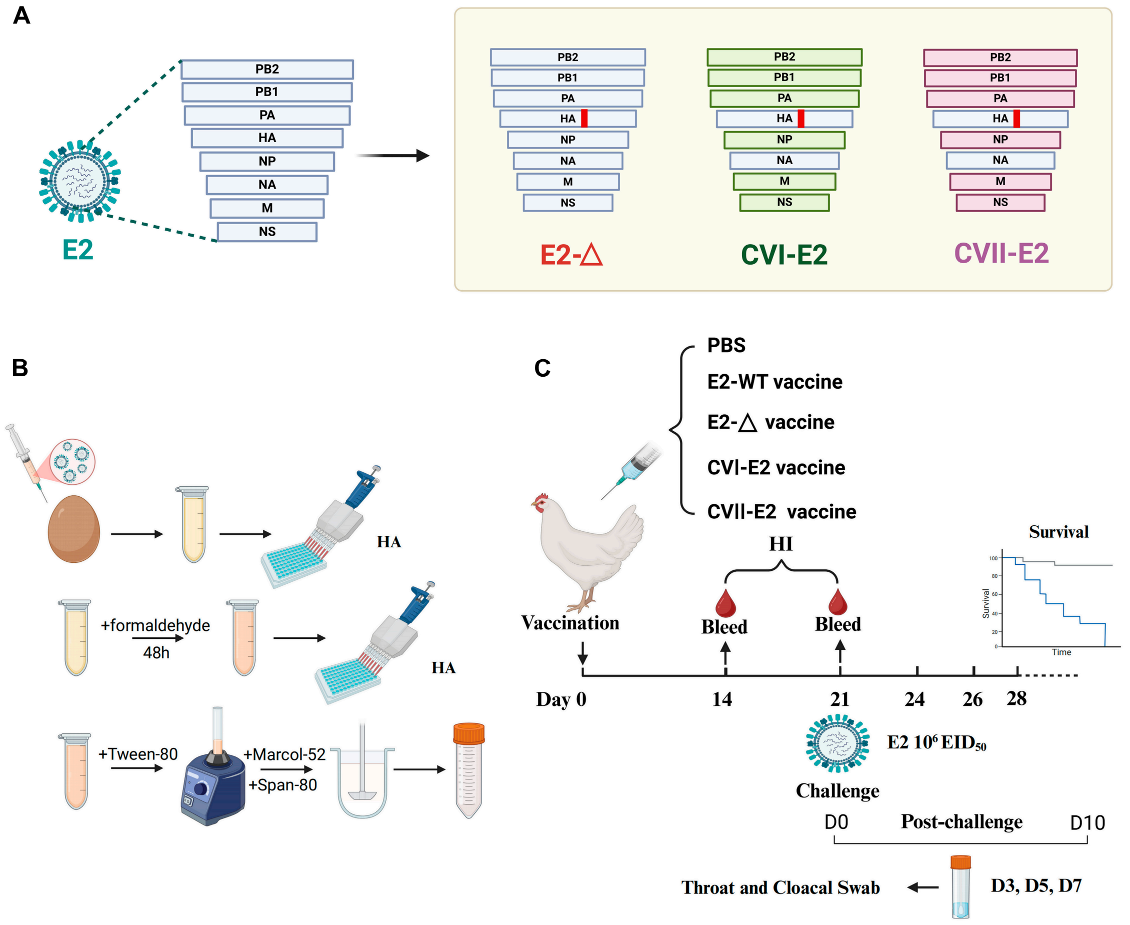


**Figure S1. Comparative Immunogenicity of Inactivated H7N9 Avian Influenza Vaccines with Different Internal Gene Backbones.**

(A) Design of recombinant H7N9 vaccine candidate strains (for E2-Δ, CVI-E2, CVII-E2). (B) Inactivated vaccine preparation procedure. (C) SPF chicken challenge protection experimental design and procedure.

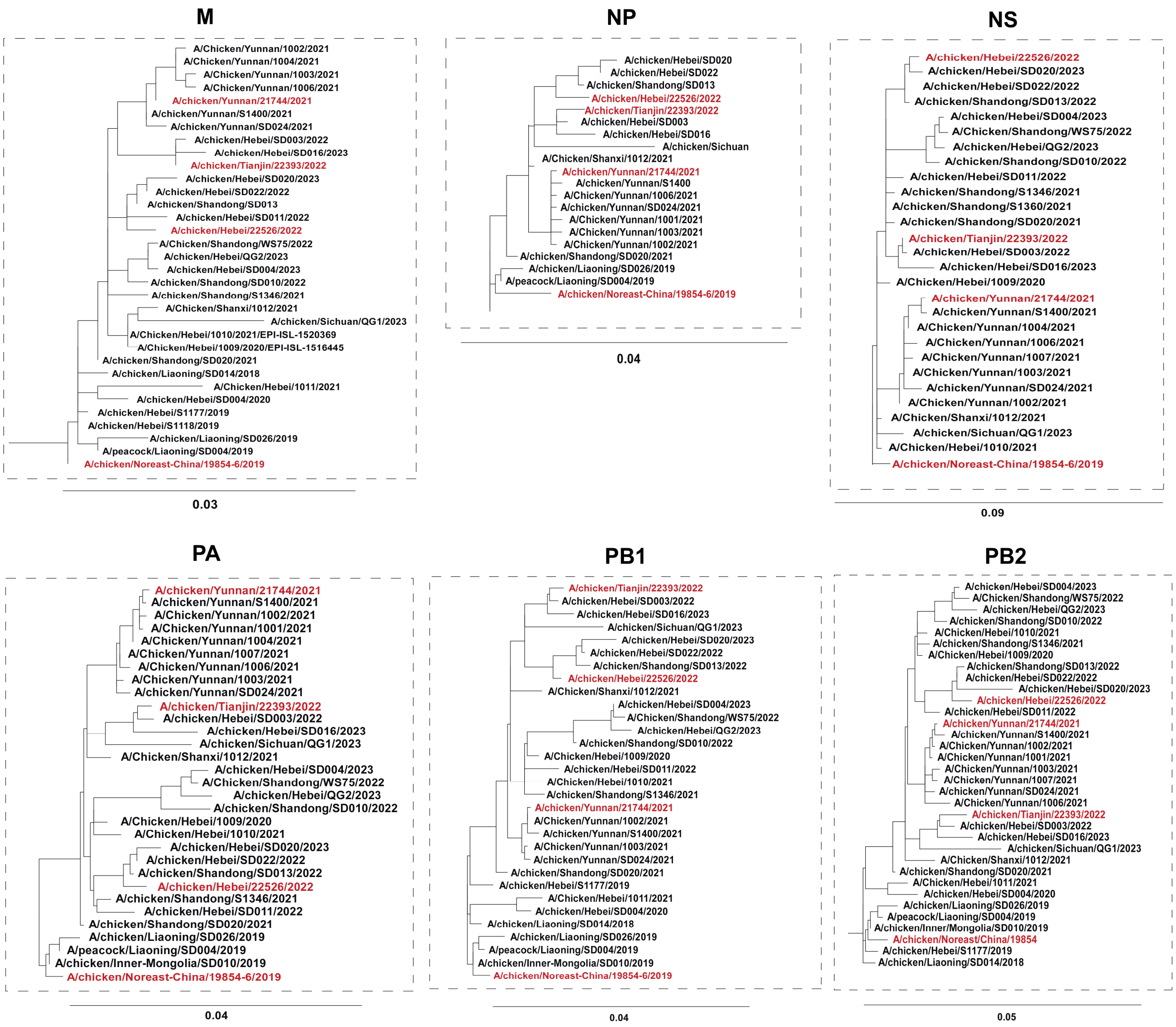


**Figure S2. Phylogenetic analysis of other genes (PB1, PB2, PA, NP, M, and NS) of H7N9 AIV (from Figure 2).**

Maximum likelihood (ML) phylogenetic trees of other genes (PB1, PB2, PA, NP, M, and NS) of H7N9 viruses. Except for the E2, 21744, 22393, and 22526 strains, all other virus sequences were downloaded from GISAID. The virus strains identified in this study are highlighted in red.

**Table S1. Primers used for the rescue of avian influenza virus by reverse genetics.**

| Primer Name | Primer Sequence（5'～3'） |
| --- | --- |
| Bm-PB2-1F | TATTCGTCTCAGGGAGCGAAAGCAGGTC |
| Bm-PB2-2341R | ATATCGTCTCGTATTAGTAGAAACAAGGTCGTTT |
| Bm-PB1-1F | TATTGGTCTCAGGGAGCAAAAGCAGGAGT |
| Bm-PB1-2341R | ATATGGTCTCGTATTAGTAGAAACAAGGAGTTTTTT |
| Bm-PA-1F | TATTCGTCTCAGGGAGCGAAAGCAGGTAC |
| Bm-PA-2233R | ATATCGTCTCGTATTAGTAGAAACAAGGTACTT |

| | |
|---|---|
| Bm-HA-F | TATTCGTCTCAGGGAGCAAAAGCAGGG |
| Bm-HA-R | ATATCGTCTCGTATTAGTAGAAACAAGGGTGTTTT |
| Bm- NP-1F | TATTCGTCTCAGGGAGCAAAAGCAGGGTA |
| Bm-NP-1565R | ATATCGTCTCGTATTAGTAGAAACAAGGGTATTTTT |
| Bm-M-1F | TATTCGTCTCAGGGAGCAAAAGCAGGTAG |
| Bm-M-1027R | ATATCGTCTCGTATTAGTAGAAACAAGGTAGTTTTT |
| Bm-NS-1F | TATTCGTCTCAGGGAGCAAAAGCAGGGTG |
| Bm-NS-890R | ATATCGTCTCGTATTAGTAGAAACAAGGGTGTTTT |
| Bm-NA-F | TATTGGTCTCAGGGAGCAAAAGCAGGAGT |
| Bm-NA-R | ATATGGTCTCGTATTAGTAGAAACAAGGAGTTTTTT |
| pHW2000-F | CTAGAGAACCCACTGCTTAC |
| pHW2000-R | TTGGGGACAGGTGTCCGTGT |
| E2-F | TGAGGTTCCAAAGGGAAGAGGCCTATTTGGTG |
| E2-R | CACCAAATAGGCCTCTTCCCTTTGGAACCTCA |

**Table S2. The accession numbers in GISAID of E2 (H7N9) strains in this study.**

| A/Chicken/Northeast China/19854-6/2019 (E2) | ACCESSION |
|---|---|
| Isolate ID | EPI_ISL_408429 |
| PB2 | EPI5277619 |
| PB1 | EPI5277620 |
| PA | EPI5277612 |
| HA | EPI1679408 |
| NP | EPI5277621 |
| NA | EPI1708359 |
| M | EPI5277622 |
| NS | EPI5277623 |

**Table S3. Viral titers in the lung tissues of SPF chickens on day 3 post-challenge in each group.**

| Group | Viral titer in lung tissues ($lgEID_{50}$/mL) (n=3) |
|---|---|
| E2-WT | 0, 0, 0 |
| E2-Δ | 0, 0, 0 |
| CVI-E2 | 0, 0, 0 |
| CVII-E2 | 0, 0, 0 |
| Control | 5.20, 5.41, 5.41 |

**Table S4. HI antibody titers of each vaccine group at weeks 2 and 3 post-immunization.**

| Group | HI titers (2 weeks post-immunization, n=10) | HI titers (3 weeks post-immunization, n=10) |
|---|---|---|
| E2-WT | 0, 1, 2, 2, 3, 3, 3, 4, 5, 5 | 6, 6, 6, 6, 6, 6, 7, 7, 7, 8 |
| E2-Δ | 2, 4, 4, 4, 4, 5, 5, 5, 6, 7 | 5, 7, 7, 8, 8, 8, 8, 8, 9, 9 |
| CVI-E2 | 0, 1, 4, 4, 4, 4, 4, 5, 5, 6 | 7, 7, 7, 7, 7, 7, 7, 7, 9, 9 |
| CVII-E2 | 0, 1, 1, 1, 1, 3, 3, 3, 4, 5 | 4, 4, 5, 5, 6, 6, 7, 7, 7, 7 |
